# Preparation and Properties of Minocycline-Loaded Carboxymethyl Chitosan Gel/Alginate Nonwovens Composite Wound Dressings

**DOI:** 10.3390/md17100575

**Published:** 2019-10-11

**Authors:** Yingjun Gao, Xing Zhang, Xiangyu Jin

**Affiliations:** Key Laboratory of Textile Science and Technology of the Ministry of Education, College of Textiles, Donghua University, Shanghai 201620, China; gaoyingj@hotmail.com (Y.G.); tulip_90@163.com (X.Z.)

**Keywords:** alginate fibers, carboxymethyl chitosan, wound dressings, antibacterial activity, drug release

## Abstract

As derivatives from marine natural biomaterials, alginate-based and chitosan-based biomaterials are commonly used in wound dressings. Calcium alginate fiber (CAF) dressings possess excellent absorption and unique gel forming performance, but the low bioactivity limits its application in wound healing. Carboxymethyl chitosan (CM-Chit) has excellent antibacterial activity, but the gel structure with weak mechanical properties restricts its application. In this study, minocycline (Mino)/CM-Chit solution was coated on the surface of plasma treated CAF needle-punched nonwovens, and then Mino loaded CM-Chit gel/CAF nonwovens composite dressings were fabricated by EDC/NHS (1-3-(3-dimethylaminopropyl)-3-ethylcarbodiimide hydrochloride/N-hydroxysuccinimide) crosslinking. The dressings had a porous composite structure, which allowed them to quickly absorb and store a large number of wound exudates. Skin-like tensile performance allowed the dressings to provide a better healing environment. Antibacterial assay against *Escherichia coli* and *Staphylococcus aureus* indicated that the addition of Mino significantly improved the antibacterial activity of the wound dressings. The tight structure of CM-Chit gel prevented the burst release of Mino so that the dressings had antibacterial activity in a certain period of release time. Cell culture assay showed that the dressings had excellent cell biocompatibility. As new functional dressings, the prepared composite dressings had excellent potential in the clinical healing of wounds.

## 1. Introduction

As the skin substitute, the ideal wound dressings should possess the following functions: (1) be a barrier to protect wound from contamination by chemicals and microorganisms; (2) allow air exchange, which provides oxygen for cell growth and excreting carbon dioxide produced by cells; (3) remove wound exudates in time and maintain the moist microenvironment between wound, wound exudates and dressings; (4) be non-toxic and non-allergenic to wound [1,2,3,4]. Nowadays, the preparation of new generation functional dressings with biological activity has become a hot research topic. These functional dressings possess various bioactivities, such as rapid hemostasis, antibacterial, anti-inflammatory, promoting cell proliferation, tissue regeneration and wound healing. The strategies of producing these functional dressings can be classified into two categories. The first is to prepare dressings directly from biologically active materials such as collagen, chitosan and alginate. The second is to add bioactive macromolecules or small molecules into the dressings, such as proteins, growth factors, drugs and even cells [5,6,7,8].

Chitosan is a cationic marine natural polysaccharide obtained from chitin after deacetylation of the N-acetyl glucosamine groups. The chemical structure of chitosan is composed by D-glucosamine and N-acetyl-D-glucosamine by β (1-4) glycosidic linkages [9]. Owing to its excellent biocompatibility and biodegradability, chitosan-based biomaterials are wildly used in biomedical fields, such as tissue engineering, drug delivery, tissue regeneration and wound healing [10,11,12,13,14,15]. The primary amine groups endow chitosan unique properties such as in situ gelation, permeation enhancement, cationic nature and antibacterial. Previous researches indicate that chitosan can inhibit the growth of bacteria and fungi and possess broad-spectrum antibiotic activity, high bacteriostatic rate. Meanwhile, chitosan has hemostatic effects and can promote the activation of macrophages and cytokines [16,17]. In the early phase of wound healing, chitosan can induce collagen synthesis and accelerate the re-epithelialization, the angiogenesis of the granular layer regeneration, which is positive for wound healing [18,19]. The application of chitosan in functional dressings has attracted increasing attention due to its antimicrobial activity and healing-promoting performance [20,21,22]. Chitosan has low solubility in water and common organic solvents, so some reagents need to be added in the preparation of chitosan-based biomaterials. Some of the reagents are bio-toxic, which limits the application of chitosan-based biomaterials in biomedicine. Carboxymethyl chitosan is a water-soluble derivative of chitosan. According to the different substitution sites of carboxymethyl groups, carboxymethyl chitosan can be divided into three types: *N*, *O*-carboxymethylated chitosan, *O*-carboxymethylated chitosan and *N*-carboxymethylated chitosan. Carboxymethyl chitosan biomaterials can be fabricated in neutral water medium, thus maintaining the activity of active substances such as small molecule drugs, proteins and cells, which expands the application range of chitosan [23,24,25,26]. Meanwhile, carboxymethyl chitosan possesses better sustained and controlled drug release performance [27,28,29,30]. Compared with chitosan, carboxymethyl chitosan has better biocompatibility, high moisture retention ability and enhanced antimicrobial property so it has better application in wound healing. The antimicrobial properties of N, O-carboxymethyl chitosan and O-carboxymethyl chitosan are better than that of chitosan. Carboxymethyl chitosan has excellent bioactivity, biodegradability, antimicrobial activity and biological affinity. It is widely used in drug delivery systems, antimicrobial dressings, tissue engineering scaffolds [31,32,33,34].

Sodium Alginate is a linear marine natural polysaccharide composed of β-D-mannuronate (M) and α-D-guluronate (G) by 1, 4-glycosidic covalent linkages. These two monomers are combined in different amount and sequential distribution to form various molecular chain fragments, such as M-blocks (MMM) G-blocks (GGG) and G-M-blocks (GMGMG) [35]. Sodium alginate possesses unique multivalent cationic chelating property. In the presence of Ca^2+^ ions, sodium alginate is synthesized into insoluble calcium alginate with “egg-box” macromolecular structure [36,37]. Thus, the materials with different structures and properties, such as micro- and nano- microfibers, hydrogels, films etc. can be produced in easy crosslinking methods. Due to the excellent biocompatibility and mechanical properties, calcium alginate fibers and its textile assembly with “tissue-like” 3D structure are extensively used in biomedical applications, such as tissue engineering scaffold, drug carriers, wound dressings [38,39,40,41,42]. The Ca^2+^ ions chelated with M groups are easily replaced by Na^+^ ions in wound exudates and then form sodium alginate hydrogels, producing moist healing conditions which are proved to reduce wound healing times [1]. The hydrogels reduce the pain severity and can be easily removed from wound without extra trauma [43]. The alginate fiber dressings are widely used in the treatment of moist chronic wounds such as diabetic foot ulcers, toxic epidermal necrolysis, necrotic leg ulcers, etc. [2,44,45]. Calcium alginate fiber dressings have limited biological activity and do not add active substances such as small molecule drugs, proteins, growth factors, which reduces its healing performance and application in wound healing.

Alginate has no ability to promote cell proliferation, migration, differentiation and specific gene expression. Meanwhile, the weak mechanical performance makes it difficult for carboxymethyl chitosan to produce a scaffold structure. In order to overcome the limitation of individual biomaterials and prepare biopolymers with multiple functions, hydrogels, microfibers, membranes and films composed by alginate and carboxymethyl chitosan have been prepared and their potential applications as wound dressings in promoting wound healing have been investigated. Compared with dressings made of individual biopolymer, these composite dressings possess better healing-promoting performance [26,46,47]. These biomaterials have excellent bioactivity, but their structures are mostly hydrogels or gels. The mechanical properties of hydrogels and gels are quite different from those of tissues and organs, which limits their application in biomedical fields, especially in wound dressings.

In this study, we developed the composite dressings made of carboxymethyl chitosan (CM-Chit) gel and calcium alginate fiber (CAF) needle-punched nonwovens. The mechanical performance, the physicochemical properties, the cell compatibility and the antibacterial activity were characterized. Meanwhile, the drug-embedded carboxymethyl chitosan-alginate composite dressings were prepared to explore the potential of the composite dressings as a drug delivery system, which endowed the composite dressings with more functions. The results evidenced that the composite dressings could be used as functional compress to accelerate wound healing.

## 2. Results and Discussion

### 2.1. Fabrication of CM-Chit Gel/CAF Nonwovens Composite Wound Dressings

**Figure 1:**
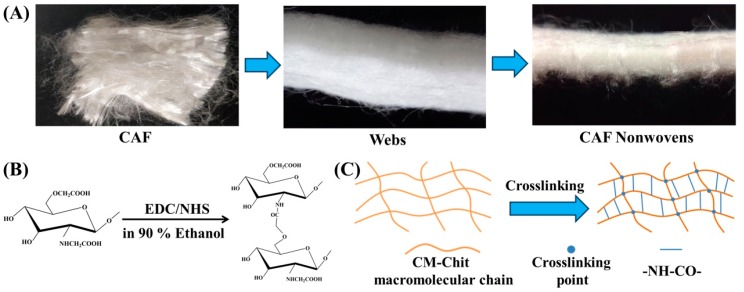
Structure changes in the fabrication of composite wound dressings. (**A**) The structure of CAF, webs and needle-punched nonwovens. (**B**) The chemical structure and (**C**) the macromolecular chains structure of CM-Chit during crosslinking.

As shown in Figure 1A, CA fibers had a certain crimp, which improved the entanglement and friction between fibers. After opening, carding and lapping, CA fibers intertwined and formed webs with a fluffy 3D structure. CA fibers were driven and moved along the thickness direction, forming fiber bundles with vertical distribution. The fiber webs were reinforced by the fiber bundles to form needle-punched nonwovens with excellent mechanical properties and porous 3D structure. As shown in Figure 1B, in the chemical crosslinking formation reaction of CM-Chit gel, EDC and NHS participated in only as intermediates but not as part of the crosslinked products. In the reaction conditions of ethanol, the *O*-isoacylurea was formed by coupling the EDC and carboxyl groups in CM-Chit macromolecular chains, and then, the amide bond was formed between the *O*-isoacylurea and the amino groups in the CM-Chit macromolecular chains, making the macromolecular chains crosslinked. The existence of NHS enhanced the stability of the crosslinked products and the residual EDC/NHS was removed after washing with Milli-Q water to reduce the biological toxicity of the composite wound dressings. As shown in Figure 1C, CM-Chit macromolecular chains distributed randomly and entangled with each other to form the solution with a certain viscosity. After chemical crosslinking reaction, the entangled macromolecular chains formed chemical bonds through amide bonds, which enhanced the interaction between macromolecular chains to form gel with stable structure.

### 2.2. Morphology and Structure Characterization

#### 2.2.1. The Rheological Behaviors and Diffusion of CM-Chit Solution

**Figure 2:**
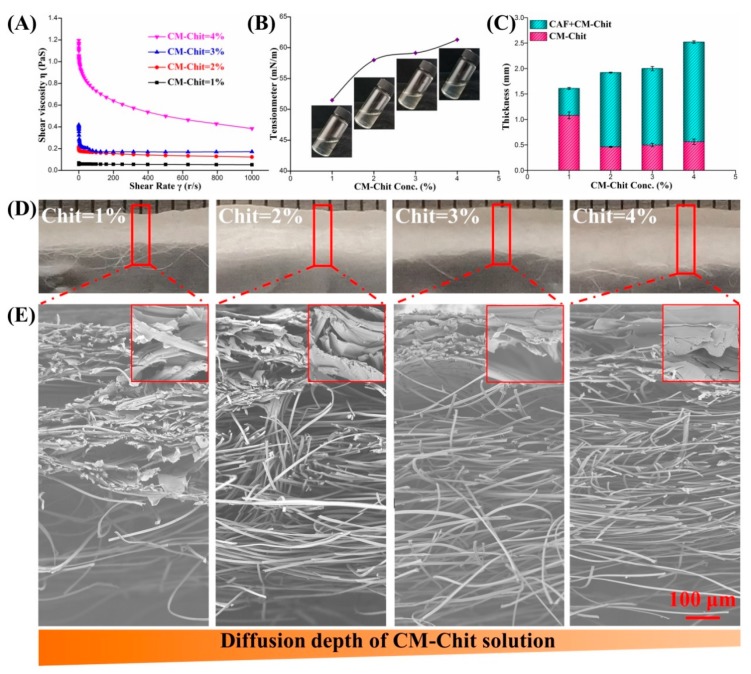
(**A**) The rheological behaviors and (**B**) the surface tension curves of CM-Chit solution with different concentrations. (**C**) The thickness of the composite wound dressings and CM-Chit gel. The cross-section morphology of the composite dressings: (**D**) The optical images and (**E**) the SEM images.

In the preparation of the composite wound dressings, the performance of solution, especially the rheological properties, greatly affects the structure and properties of dressings. The rheological properties of CM-Chit solution were shown in Figure 2A. As the CM-Chit concentration increased from 1% (*w*/*v*) to 4% (*w*/*v*), the solution viscosity improved from 0.05 PA s to 1.20 PA s. With the increase of macromolecules content in solution, the entanglement degree and interaction force between macromolecular chains increased. Especially when the solution concentration increased from 3% (*w*/*v*) to 4% (*w*/*v*), the solution viscosity increased greatly. When solution was sheared, the entanglement between macromolecular chains was destroyed, reducing the viscosity of the solution. With the increase of the shear rate, the degree of disruption of intermolecular entanglement increased. The effect of shear rate on the viscosity of solution with high concentrations solution was significantly higher than that of solution with low concentrations. When the shear rate was higher than 20 r/s, the viscosity of solution with high concentrations decreased obviously and tended to be stable. The surface tension was another important indicator of solution properties, which was closely related to the density and viscosity of solution. As shown in Figure 2B, with the increase of solution concentration and viscosity, the surface tension of CM-Chit solution increased significantly. The large surface tension of the solution with high concentrations improved the force needed to surmount the spontaneous contraction, which significantly reduced the fluidity of CM-Chit solution and the diffusion of the solution to CAF nonwovens. As shown in Figure 2C, when the CM-Chit solution concentration was low, the tight composite structure reduced the thickness of the composite wound dressings. The high fluidity and viscosity of the solution with low concentrations promoted the diffusion of the solution into the nonwovens, making the gel diffusion depth reach 70% of the thickness of the composite dressings. With the increase of concentration, the diffusion depth of CM-Chit solution into CAF nonwovens declined, which made the thickness of CM-Chit gel increase and the thickness of the composite dressings decline significantly.

#### 2.2.2. The Cross-Section Morphology and Structure Characterization

The optical cross-section morphology of the composite wound dressings was shown in Figure 2D. The composite wound dressings possessed porous structure composed of gel and nonwovens. The porous structure was divided into three parts: CM-Chit gel, gel/nonwovens composite and nonwovens. As the diffusion of CM-Chit solution decreased, the boundary between gel and nonwovens became obvious. The cross-section SEM images of the composite dressings (Figure 2E) showed that when the CM-Chit solution concentration was low, the solution penetrated into the CAF nonwovens and diffused along the fibers and the pores in the nonwovens. As the concentration increased, the penetration of CM-Chit solution along the thickness of CAF nonwovens and the diffusion along fibers and pores decreased, which reduced the composite structure and weakened the structural change of CAF nonwovens.

#### 2.2.3. The Surface Morphology and Structure Characterization

**Figure 3:**
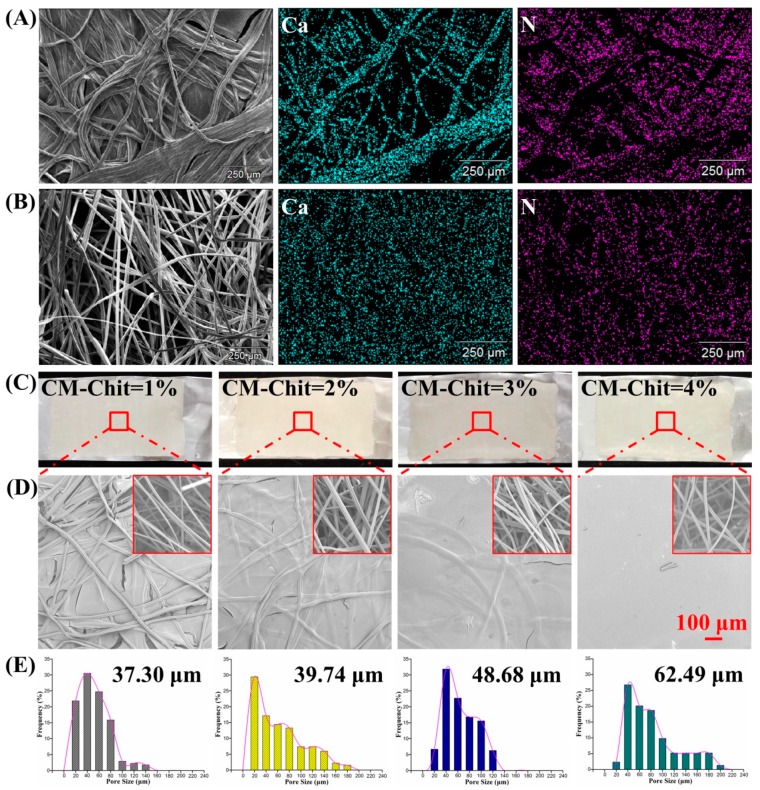
EDX mapping images of (**A**) the CM-Chit gel surface, (**B**) the CAF nonwovens surface. The surface morphology of the composite dressings: (**C**) the optical images, (**D**) the SEM images. (**E**) The pore size and its distribution of the composite dressings.

As shown in Figure 3A,B, energy dispersive X-ray (EDX) scanning was used to observe the distribution of characteristic elements on the CM-Chit gel surface and CAF nonwovens surface. The distribution of CM-Chit gel was characterized by N element content, meanwhile the distribution of CA fibers was characterized by Ca element content. On the surface of CM-Chit gel, a large number of N elements were distributed, with a content of 9.16%. The content of Ca was 5.01%, and the relationship between the distribution of N and Ca was complementary. On the surface of CAF nonwovens, the Ca element was evenly distributed with a content of 15.49%. Due to a large number of pores between CA fibers, the N element distributed on the surface of CAF nonwovens was detected with a relatively low content, which was 1.07%.

Figure 3C,D showed the surface morphology of the composite dressings. When the amount of CM-Chit solution penetrated into CAF nonwovens was high, the obvious fibrous structure was observed on the CM-Chit gel surface, which separated the CM-Chit gel structure into different regions. When the amount of CM-Chit solution diffused into CAF nonwovens declined, the fibers distributed on the gel surface gradually decreased and eventually disappeared. The CM-Chit gel interacted and formed a compact surface structure.

As shown in Figure 3E, with the increase of CM-Chit solution concentration from 1% (*w*/*v*) to 4% (*w*/*v*), the mean pore size of the composite dressings gradually increased from 37.30 μm to 62.49 μm. The main distribution ranges of pore size also changed significantly. The main distribution range of pore size of the composite dressings prepared by CM-Chit solution with concentrations of 1% (*w*/*v*), 2% (*w*/*v*), 3% (*w*/*v*) and 4% (*w*/*v*) were 20–60 μm, 20–80 μm, 40–100 μm and 40–120 μm, respectively. With the increase of CM-Chit solution concentration, the number of pores with large size increased significantly. This was mainly because that with the decrease of CM-Chit permeability, the destruction of CAF nonwovens porous structure caused by CM-Chit gel structure was reduced, thus increasing the distribution of large pores in the composite dressings.

### 2.3. The Properties Characterization

#### 2.3.1. The Chemical Structure Characterization

**Figure 4:**
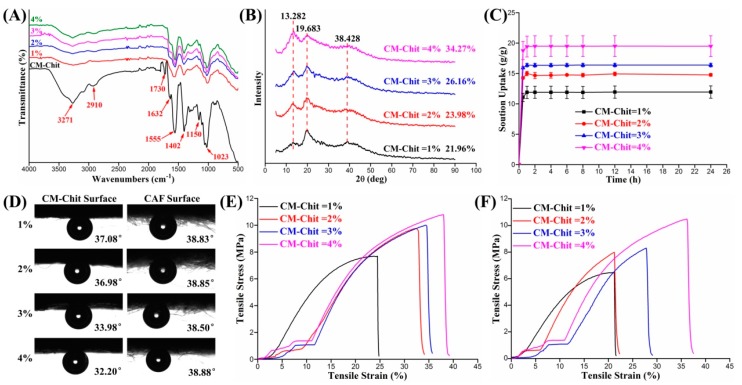
Characterization of physicochemical properties of the composite wound dressings: (**A**) The FTIR spectrum. (**B**) The XRD spectrum. (**C**) The solution uptake. (**D**) The contact angels. (**E**) The strain-stress curves in MD. (**F**) The strain-stress curves in CD.

The chemical structure of the composite dressings was characterized by FTIR and XRD spectra. The functional groups structure of the composite dressings before and after crosslinking was shown in Figure 4A. From the FTIR spectra of CM-Chit, the absorption peaks at 3271 cm^−1^, 2910 cm^−1^ and 1730 cm^−1^ were the characteristic peaks of O-H, C-H and -COOH groups, respectively. The absorption peaks at 1632 cm^−1^, 1555 cm^−1^ and 1402 cm^−1^ characterized the COO-, N-H and -CH_2_COOH groups, which indicated the transition of amino and hydroxyl groups in chitosan macromolecules caused by carboxymethylation. From the FTIR spectra of the crosslinked CM-Chit, the stretching vibration peaks of -COOH groups at 1730 cm^−1^ disappeared, and the absorption peaks of macromolecular chains at 1632 cm^−1^ and 1402 cm^−1^ decreased, indicating the decrease of carboxymethyl groups linked with amino groups. The characteristic peaks at 1550 cm^−1^, 1407 cm^−1^ and 1025 cm^−1^ moved to 1555 cm^−1^, 1402cm^−1^ and 1023cm^−1^, indicating the changes of N-H, C=O, C-N and other groups. With the increase of CM-Chit concentration, the number of macromolecular chains involved in crosslinking increased, and the peak strength of characteristic absorption peaks of -COOH group decreased gradually; meanwhile, the peak strength of characteristic absorption peaks of N-H and C-H groups increased gradually. After crosslinking, the interconnected macromolecular chains formed a compact aggregated structure. With the increase of CM-Chit concentration, the degree of entanglement and crosslinking between macromolecular chains increased, resulting in a significant increase in the crystal structure and the intensity of the diffraction peaks at 2θ = 13.282° and 2θ = 38.428°. The crystallinities of the composite dressings fabricated by CM-Chit solution with concentration of 1% (*w*/*v*), 2% (*w*/*v*), 3% (*w*/*v*) and 4% (*w*/*v*) were calculated as 21.96%, 23.98%, 26.16% and 34.27%, respectively.

#### 2.3.2. The Hydrophilic Properties Characterization

The solution absorption performance of the composite dressings was shown in Figure 4C. The composite dressings absorbed solution quickly and reached the maximum uptake ratio in a short time and kept stable. With the increase of CM-Chit solution concentration, the solution absorption of the composite dressings increased significantly. CM-Chit gel and CA fibers contain a large number of polar groups, which makes them have excellent absorption performance [33,35]. Owing to the porous structure of the composite dressings, the solution absorbed by the composite dressings was divided into four parts: the solution absorbed by CM-Chit gel, the solution absorbed by CA fiber, the solution distributed in the composite structure and the solution distributed in the pores of CAF nonwoven. When the CM-Chit concentration was low, the high proportion of CM-Chit gel encapsulated the fibers, reducing the amount of solution absorbed by the pores of CAF nonwovens. The solution mainly distributed in the CM-Chit gel and fibers; meanwhile, the smaller pore size and porosity resulted in lower solution absorption and storage capacity. For the composite dressings fabricated by CM-Chit with high concentration, the large pore size and high porosity proportion improved the solution absorption and storage properties of the composite dressings.

#### 2.3.3. The Contact Angles

The contact angles of the surface of CM-Chit gel and CAF nonwovens were illustrated in Figure 4D. For hydrophilic materials, the contact angle decreases with the increase of absorbency [48,49]. The contact angle of CM-Chit gel surface was smaller than that of CAF nonwovens surface, indicating that the CM-Chit gel possessed better hydrophilicity. The increase of CM-Chit concentration significantly improved the hydrophilicity of the gel. When the concentration increased from 1% (*w*/*v*) to 4% (*w*/*v*), CM-Chit solution crosslinked and formed a homogeneous gel surface without CA fibers distribution, reducing the contact angle from 37.08° to 32.20°. Due to the structure of CAF, nonwovens surface was stable, and the contact angle of CAF nonwovens did not change with the increase of CM-Chit solution concentration.

#### 2.3.4. Tensile Mechanical Properties Testing

The tensile properties of the composite dressings were shown in Figure 4E,F. The tensile strengths of the composite dressings fabricated by CM-Chit solution with different concentrations were in the range of 6–12 MPa with strain range of 20%–40%. It was in the range of tensile mechanical properties of healthy skin tissue (tensile strength: 1–20 MPa, tensile strain: 30%–70%) [50]. Therefore, the composite dressings had excellent biomechanical compliance to use as a substitute for healthy skin in the clinical treatment of wounds. Compared with the tensile curves of nonwovens, the tensile process of the composite dressings was more complicated due to the existence of composite structure, which was divided into three stages. The first stage was the CM-Chit gel stretching stage. At this stage, the gel was stretched along the tensile direction, and the interaction between macromolecule chains was the main force. The second stage was the straightening stage of CA fibers. At this stage, the CM-Chit gel fractured and the interactions between fibers were the main force. With the rearrangement and stretching of a large amount of fibers, the tensile strength of the composite dressings increased significantly. The third stage was the stretching stage of CAF nonwovens, in which the entanglement force, cohesion force and friction force between CAF fibers were the main mechanical action forms. With the increase of CM-Chit concentration, tighter entanglement and crystalline structure of macromolecule chains improved the tensile strength and strain of the gel at the first stage. The increase of the proportion of CAF nonwovens enhanced the tensile mechanical performance of the composite dressings fabricated with high CM-Chit concentration.

### 2.4. Drug Release and in Vitro Antibacterial Activity of Mino-Loaded Composite Dressings

#### 2.4.1. The Loading and Release of Mino

**Figure 5:**
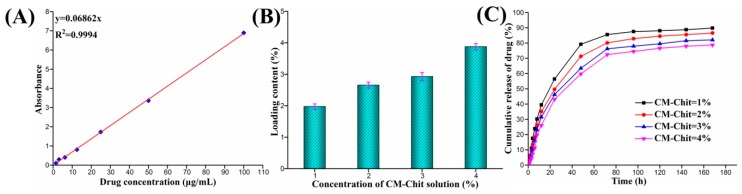
Drug loading and release assay: (**A**) Standard curve the drug concentration. (**B**) The drug loading content, (**C**) the drug release curves of CM-Chit gel with different concentrations.

The absorbance of Mino-PBS solutions with concentrations of 100, 50, 25, 12.5, 6.25, 3.125 and 1.5625 ug/mL at 272 nm was measured by ultraviolet spectrophotometer. The standard curve of Mino concentration (C as y) and absorbance (A as x) was drawn and the standard equation was calculated (shown in Figure 5A). The standard absorption equation of Mino was A = 0.06862C. As shown in Figure 5B, when the concentration of CM-Chit solution increased from 1% (*w*/*v*) to 4% (*w*/*v*), the Mino loading rate of CM-Chit gel increased from 53% to 79.5%, indicating that tight macromolecular chains entanglement structure facilitated the drug loading.

The Mino release profile from the Mino loaded composite dressings was investigated and the release curves were illustrated in Figure 5C. In the initial 4 h, the cumulative release amounts of CM-Chit gel with different concentrations were 17.5%, 13.5%, 10% and 7%, respectively, which indicated that CM-Chit gel avoided drug burst release. Subsequent to the initial fast drug release, the drug release became sustained and gentle. When the release time was 168 h, the cumulative drug release amounts were 90%, 86.5%, 82 % and 79%, respectively. The concentration of CM-Chit slightly affected the drug release profile loaded in the composite dressings. At the same release time, the cumulative drug release amount declined with the increase of CM-Chit concentration. The drug release from the composite dressings fabricated by low CM-Chit concentration reached the dynamic equilibrium quickly. For the composite dressings fabricated by CM-Chit of a concentration of 4% (*w*/*v*), the drug release reached the dynamic balance at 120 h. The high CM-Chit concentration and tight gel network made it difficult for the loaded drugs to penetrate into the solution, thus prolonging the time of drug release and the effective drug concentration.

#### 2.4.2. In Vitro Antibacterial Activity Assay

**Figure 6:**
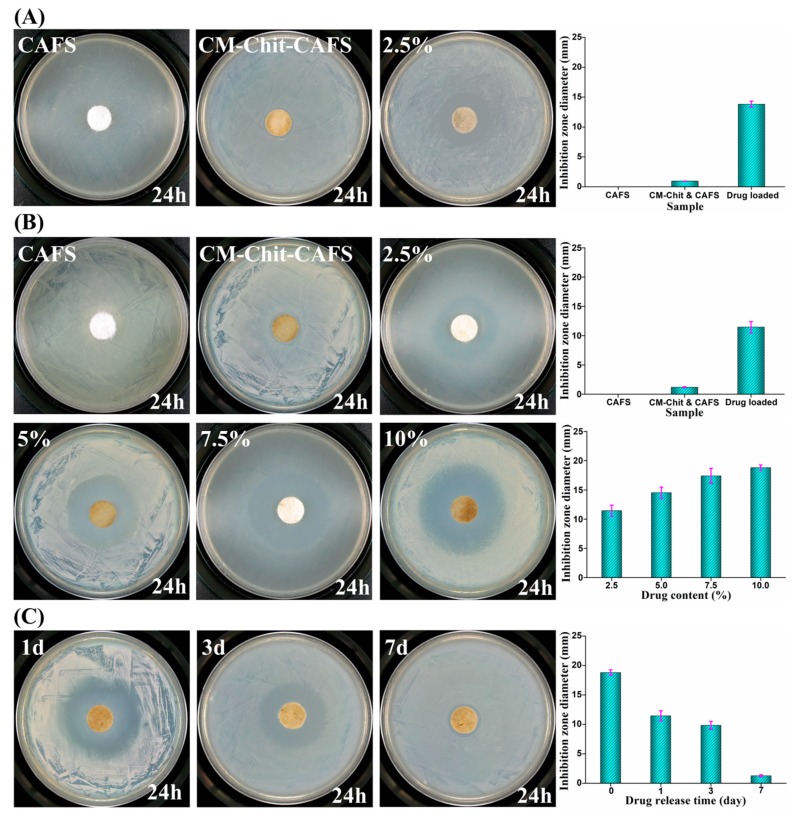
The antibacterial activity of CAF nonwovens, the composite dressings and the Mino loaded composite wound dressings (**A**) against *E. coli* (**B**) against *S. aureus*. (**C**) The antibacterial activity of Mino loaded composite dressings released for 1, 3, 7 days against *S. aureus*.

The loaded Mino was expected to improve the antimicrobial activity of the composite dressings. The antibacterial activity of CAF nonwovens, composite dressings and Mino loaded composite dressings were evaluated by inhibition zone after 24 h of bacterial culture. As shown in Figure 6A,B, CAF nonwovens had no antimicrobial activity and did not inhibit the apparent growth of *Escherichia coli (E. coli) and Staphylococcus aureus* (*S. aureus*). The positive charge in CM-Chit macromolecules interacts with the negative charge on the surface of bacteria, destroying the cell membrane of bacteria and causing cell death [31]. The widths of inhibition zones of the composite dressings against *E. coli* and *S. aureus* were 0.95 mm and 1.2 mm, respectively. Therefore, the composite dressings possessed slight antimicrobial activity against *E. coli* and *S. aureus*. When the composite dressings contained Mino with a low concentration of 2.5% (*w*/*v*), the widths of inhibition zones against *E. coli* and *S. aureus* expanded to 13.82 mm and 11.45 mm, respectively. The antimicrobial activity of the composite dressings was significantly improved by the loading of Mino, meanwhile the width of inhibition zone against *S. aureus* expanded to 18.8 mm with the Mino concentration increased to 10% (*w*/*v*).

The composite dressings loaded with 10% (*w*/*v*) Mino were immersed in PBS to release. The antibacterial activity against *S. aureus* of the Mino loaded composite dressings (4% (*w*/*v*) CM-Chit, 10% (*w*/*v*) Mino) after 1, 3, 7 days release was investigated and illustrated in Figure 6C. With the release of drug, the width of the inhibition zone and the antimicrobial activity of Mino loaded composite dressings decreased gradually. The widths of the inhibition zone of the Mino loaded composite dressings were 11.84 mm and 9.84 mm, respectively after 1 and 3 days of release, indicating that the Mino loaded composite dressings still had obvious antibacterial activity. After 7 days of release, the inhibition zone of the drug loaded composite dressings was only 6.75% of the inhibition zone of the unreleased Mino loaded composite dressings, but its antibacterial activity was still better than those of the composite dressings and CAF nonwovens. The results indicated that Mino in the composite dressings maintained certain antimicrobial activity and effective antimicrobial concentration in a certain period of time, which inhibited the growth of bacteria.

### 2.5. HUVECs Proliferation and Spreading on the Composite Dressings

**Figure 7:**
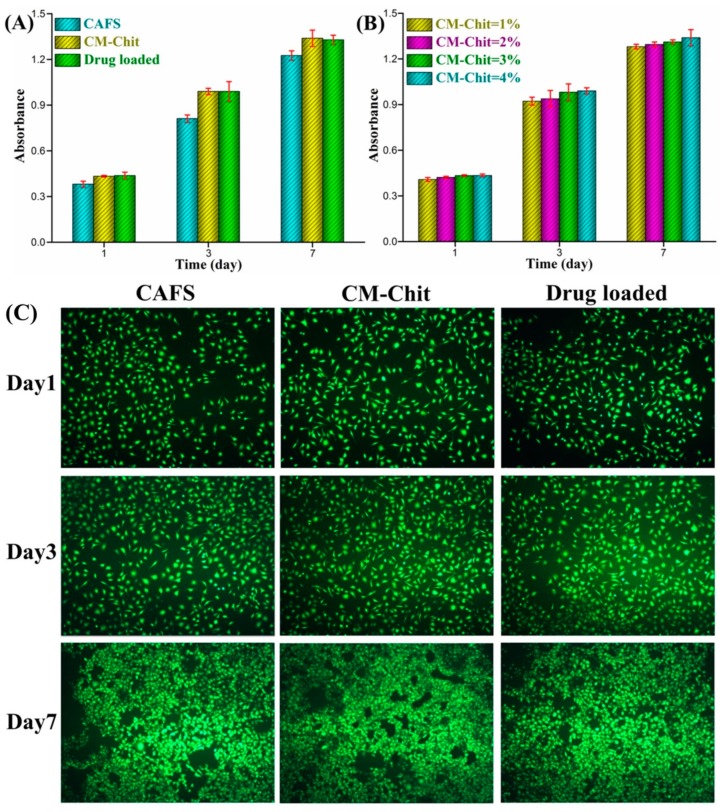
Cell Cytotoxicity of the composite wound dressings: (**A**) The proliferation of cells cultured on different dressings. (**B**) The proliferation of cells cultured on the CM-Chit gel surface. (**C**) The confocal laser scanning microscope (CLSM) images of the cells cultured on the CAF nonwovens, CM-Chit gel surface of the composite dressings and the drug loaded composite dressings.

The cell cytotoxicity of CAF nonwovens, composite dressings and Mino loaded composite dressings was evaluated by studying the growth and proliferation of HUVECs on different dressings. Figure 7A illustrated the CCK-8 assay absorbance of cells cultured on CAF nonwovens, composite dressings with 4% (*w*/*v*) CM-Chit and composite dressings with 10% (*w*/*v*) Mino. With the increase of incubation time, the numbers of cells cultured on the three dressings increased significantly, showing that the fabricated dressings possessed better biocompatibility. The absorbance of cells cultured on the CM-Chit gel surface of the composite dressings was slightly higher than that of cells cultured on CAF nonwovens, which indicated that the composite dressings had better biocompatibility. Moreover, with the increase of CM-Chit concentration, the absorbance of cells cultured on the gel surface increased slightly (Figure 7B). The previous research showed that Mino had no toxicity to cells [51]. The absorbance of cells cultured on the surface of the Mino loaded composite dressings was not significantly different from that of cells cultured on the surface of the composite dressings. Therefore, the loading of Mino did not affect the growth and proliferation of cells on the surface of the composite dressings.

Figure 7C showed stained live cells CLSM images to characterize the morphology and spreading of cells cultured on the dressings. The cells attached to the dressings with good morphology. Due to the proliferation and spreading of cells, the amount and the distribution range of cells increased significantly over the incubation time. Compared with the rough surface structure of CAF nonwovens, the smooth CM-Chit gel structure of the composite dressings provided better spreading conditions for cells. Therefore, cells cultured on CM-Chit gel surface of the composite dressings had better growth and proliferation activity.

## 3. Materials and Methods

### 3.1. Materials

The CAF (3.01 dtex ± 0.03 dtex, 50 mm) was provided from United Medical Technologies Co., Ltd. (Foshan, China). Carboxymethyl chitosan (CM-Chit) was purchased from Yuanye Bio-Technology Co., Ltd. (Shanghai, China). Its degree of deacetylation and degree of substitution were 85% and 80% respectively. 1-3-(3-dimethylaminopropyl)-3-ethylcarbodiimide hydrochloride (EDC), N-hydroxysuccinimide (NHS) and minocycline hydrochloride were purchased from Sigma-Aldrich (St. Louis, MO, USA). Ethyl alcohol and sodium chloride were purchased from Aladdin (Shanghai, China). Reagents used in bacterial culture assay (yeast extract, tryptone, agar, etc.) were obtained from Thermo Scientific Oxoid (Basingstoke, UK). Reagents used in cell culture in vitro were purchased from Gibco (Thermo Fisher Scientific Co., Waltham, MA, USA). Cell Counting Kit (CCK8) was purchased from Gibco (Thermo Fisher Scientific Co., Waltham, MA, USA). Cell viability (Calcein-AM/PI) test kit was procured from KeyGEN BioTECH Co. (Nanjing, China). The water purified by a Milli-Q water purification system (Millipore, Bedford, MA, USA) was used in all the experiments and the resistivity higher than 18.2 MΩ.

### 3.2. Fabrication of CM-Chit Gel/CAF Nonwovens Composite Wound Dressings

**Figure 8:**
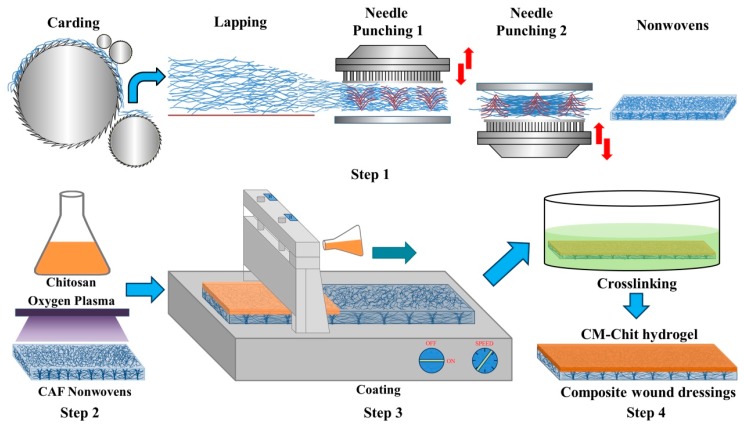
Schematic diagram of fabrication process of CM-Chit gel/CAF nonwovens composite wound dressings.

As shown in Figure 8, the process of fabrication for CM-Chit gel/CAF nonwovens composite wound dressings was divided into four steps. Step 1: Fabrication of CAF nonwovens. Calcium alginate fibers were opened and carded into fiber webs and then the webs were cross-lapped and needle-punched, forming the needle-punched nonwovens with a porous 3D structure and a surface density of 120g/m^2^. Step 2: CM-Chit solution preparation and plasma treatment of CAF nonwovens. CM-Chit was dissolved in Milli-Q water and stirred to form a uniform solution. The fluidity and surface tension of CM-Chit solution determined the diffusion of the solution in nonwovens. The rheological behaviors of CM-Chit solution were measured by ARES rheometers (TA Instruments Co., New Castle, DE, USA). The surface tension of CM-Chit solution was measured and characterized by the surface tensiometer (DCAT11, Data Physics Instruments Co., Filderstadt, Germany). In order to improve the affinity of CAF nonwovens and the diffusion of CM-Chit solution in nonwovens, surface oxygen plasma treatment on CAF nonwovens was carried out by low-temperature plasma treatment instrument (HD-300, Zhongke ChangTsi Plasma Processing Apparatus Plasma Technology Co., Changzhou, China), the treatment time was set as 20 s. Step 3: CM-Chit solution coating. CM-Chit solution was coated on the surface of CAF nonwovens using a small laboratory coating machine (MSK-AFA-Ⅲ, Milliren Technologies, Inc., Newburyport, MA, USA). The running speed of the scraper was set as 15 mm/s, and the distance between the scraper and the surface of CAF nonwovens was set as 1 mm. Step 4: CM-Chit crosslinking. CAF nonwovens coated with CM-Chit solution was immersed in EDC/NHS solution (90% ethanol and 10% water) and placed at room temperature for crosslinking. After 24 h, the samples were taken out and washed with Milli-Q water for 3 times to remove excess EDC/NHS and ethanol.

### 3.3. Morphology and Porous Structure Characterization

The surface and cross-section morphology of the lyophilized composite wound dressings were observed by a scanning electron microscope (TM3000, Hitachi, Tokyo, Japan) at an acceleration voltage of 10kV. The porous structure of composite wound dressings, including the pore size and it distribution, was measured using the capillary flow porosimetry (CFP, Porometer 3G zh; Quantachrome Instruments, Boynton Beach, FL, USA).

### 3.4. Macromolecular Structure Characterization

The macromolecular structure of composite wound dressings was characterized by Fourier Transform Infrared (FTIR) and X-ray diffraction (XRD). The FTIR spectra were measured by Nicolet 6700 FTIR spectroscopy (Thermo Fisher Scientific, Waltham, MA, USA) in the attenuated total reflection mode (ATR). The absorption spectral range was 500–4000 cm^−1^. The crystal and aggregation structure of CM-Chit and CAF macromolecules were investigated by the D/max RB X-ray diffractometer (Rigaku Co., Tokyo, Japan) with Nickel-filtered Cu kα radiation in the 2*θ* range of 0–60°.

### 3.5. Solution Absorption Performance Testing

The hydrophilicity of the composite wound dressings can be characterized by the contact angle. The contact angles the different surfaces of composite wound dressings were measured by the Contact Angle Tester (OCA15EC; Data Physics Instruments Co., Filderstadt, Germany). The samples (5 cm ×5cm, weight as *W_0_*) were immersed in Milli-Q water at 37 °C for 0.5, 1, 2, 4, 6, 8, 12, 24 h. The samples after absorption of water were weighed after removing the residual water (*W_1_*). The solution uptake rate (*g/g*) was calculated by (*W_1_ − W_0_*)/*W_0_*.

### 3.6. Tensile Mechanical Performance Testing

The tensile mechanical performance of the composite wound dressings was measured by an electromechanical universal testing machine (WDW-20; Shanghai Hualong Test Instruments Co., Shanghai, China) with a gauge length of 10 cm and a stretching speed of 50 mm/min.

### 3.7. Mino Loading and in Vitro Release

#### 3.7.1. Fabrication of Drug Loaded CM-Chit gel/CAF Nonwovens Composite Wound Dressings

For drug loading, Mino was dissolved to CM-Chit solution and the mixture was stirred at room temperature in dark conditions to form uniform Mino/CM-Chit solution, and then Mino loaded CM-Chit gel/CAF nonwovens composite wound dressings were fabricated as described in Section 3.2 in dark conditions. The crosslinking solution and washing solution were collected and measured by a UV spectrophotometer (JASCO V530, JASCO, Tokyo, Japan) at 272 nm. The Mino loading content (Lc) was calculated according to Equation (1):

(1) $$\mathrm{Loading}\;\mathrm{content}\;=\;\left(1-\frac{C\times V}{m\;}\right)\times 100\%$$

Where *C* was the drug concentration of the collected solution; *V* was the volume of the collected solution; *m* was the weight of Mino in CM-Chit solution.

#### 3.7.2. In Vitro Mino Release

The drug release from the Mino loaded CM-Chit gel/CAF nonwovens composite wound dressings was investigated in accordance with the methods described in the previous research [52]. A total of 1g of each sample was immersed in centrifuge tube with 100 mL PBS solution (pH = 7.4) and placed in a shaker with a constant temperature of 37 °C and a speed of 100 rpm. After the selected time interval of release, 5 mL of the released solution was taken out and measured by a UV spectrophotometer at 272 nm. In order to ensure the uniformity of the release solution, 5 mL fresh PBS solution was added after each extraction. The concentration of the released solution was calculated according to the standard calibration of Mion in PBS solution. The cumulative release ratio of Mino was calculated according to Equation (2):

(2) $$Q\;(\%)\;=\;\frac{C_{n\cdot \;}V+V_{i}\sum_{i=0}^{n-i}C_{i}}{m_{d}}$$

Where, Q was the cumulative release ratio of Mino; *n* was number of displacement release solution; *Cn* was the concentration of drug in the release solution at nth release time interval, mg/mL; *V* was the volume of the release solution; *Vi* was the volume of drug release solution at the ith release time interval; *Ci* was the concentration of drug in the release solution at ith release time interval, mg/mL; *V_0_* and *C_0_* were 0; *m_d_* was the quality of drug in sample.

### 3.8. In Vitro Antibacterial Activity Assay

The antibacterial activity of the fabricated wound dressings was evaluated by the agar plate diffusion method. *Escherichia coli (E. coli),* a gram-positive bacterium, and *Staphylococcus aureus (S. aureus),* a gram-positive bacterium, were used in this assay. The frozen *E. coli and S. aureus* were activated and cultured in the trypticase soy broth (TSB) medium in an oscillation incubator with a temperature of 37 °C and a speed of 120 rpm. The bacterium/TSB suspension was spread on the agar plates and the sterilized circular samples (n = 3) with a diameter of 14 mm were placed on the spread bacterium/TSB suspension. The agar plates were incubated in a constant temperature incubator with a temperature of 37 °C. After 24 h, the diameter of the inhibition zone was calculated from the images of agar plates captured by the automatic colony counter (Hangzhou Shineso Science & Technology, Hangzhou, China). The width of inhibition zone (H) was calculated according to Equation (3):

(3) $$H\;=\;\frac{Outer\;diameter\;of\;the\;inhibition\;zone-14\;mm}{2}$$

The Mino loaded wound dressings samples were placed in PBS for drug release. After 1, 3, 7 days, the antimicrobial activity of the samples was measured using the same method.

### 3.9. In Vitro Cell Cytotoxicity Assay

#### 3.9.1. Cell Culture

Human umbilical vein endothelial cells (HUVECs, Cell Bank of the Chinese Academy of Science, Shanghai, China) were cultured in medium with 90% (*v*/*v*) high glucose DMEM, 10% (*v*/*v*) fetal bovine serum (FBS) and 1% (*v*/*v*) penicillin/streptomycin at 37 °C and 5% CO_2_. The sterilized wound dressings were placed in 24-well cell culture plates. The HUVECs (10^5^ cells/well) were seeded on the samples and cultured in medium at 37 °C and 5% CO_2_.

#### 3.9.2. Cell Proliferation Assay

The proliferation of cells on the wound dressings was measured by CCK-8 assay. After 1, 3 and 7 days of culture, the medium was removed and 400 μL CCK-8 test solution was added into the well and cultured in dark conditions at 37 °C and 5% CO_2_. After 4 h of incubation, the absorbance value (O.D. Values) at 450 nm of the supernatant in each well was measured by a microplate reader (Multiskan GO, Thermo, Waltham, MA, USA).

#### 3.9.3. Cell Morphology

After 1, 3 and 7 days of culture, the medium was removed and the cells were washed by PBS for 3 times. Calcein-AM working solution was added to stain the cells and incubated at 37 °C and 5% CO_2_. After 1 h, the working solution was removed and the stained cells were washed by PBS and then visualized by confocal laser scanning microscope (CLSM, LSM 700, Carl Zeiss, Oberkochen, Germany).

## 4. Conclusions

In this study, minocycline/carboxymethyl chitosan solution was coated on the surface of plasma treated calcium alginate fibers needle-punched nonwovens and then immersed in EDC/NHS crosslinking solution. CM-Chit was crosslinked to form gel, thereby Mino loaded CM-Chit gel/CAF nonwovens composite dressings were fabricated. SEM images showed that the composite dressings had a porous structure, which was composed of gel, gel/nonwovens composite structure and nonwovens porous structure. With the increase of CM-Chit solution concentration, the thickness of gel structure and the pore size distribution was changed. The dressings had excellent absorption performance and quickly reached the maximum absorption volume so that wound exudates could be quickly transferred from the wound. The excellent mechanical properties of CAF nonwovens greatly improved the mechanical properties of CM-Chit gel so that the composite dressings had similar mechanical properties to skin. The addition of Mino significantly increased the antimicrobial activity of the composite dressings, and the gel structure avoided the burst release of the drug, which enabled the drug in the effective concentration within a certain period of time to achieve the antibacterial effect. The cells cultured on the composite dressings had good growth and proliferation activity. As new functional dressings, due to the excellent absorption performance, skin-like mechanical properties, long-term antimicrobial activity, drug sustained-release properties and biocompatibility, the prepared composite dressings had great potential in wound healing.
